# Hospitality Work as Social Reproduction: Embodied and Emotional Labour during COVID-19

**DOI:** 10.1177/00380385231189190

**Published:** 2023-09-20

**Authors:** Charlotte Jones, Lauren White, Tig Slater, Jill Pluquailec

**Affiliations:** Swansea University, UK; University of Exeter, UK; University of Sheffield, UK; Sheffield Hallam University, UK; Sheffield Hallam University, UK

**Keywords:** body work, cleaning, COVID, emotional labour, employment, hospitality, hygiene, pandemic, social reproduction, work

## Abstract

This article focuses on how the imaginary of a ‘safe’ environment was visualised and conveyed within the hospitality sector during the COVID-19 pandemic, drawing on diaries and interviews with 21 workers in the UK. Our findings show increased workloads for hospitality staff, compounded by anxieties of risk and individualised COVID-19 regulation work. This includes workers’ negotiations of corporeal boundaries and distancing from customers, the visible cleaning of communal areas and recuperation and care work for their own bodies and others in shared living spaces. We draw on conceptualisations of embodied and emotional labour to understand these experiences, reflecting on the importance of the actions performed by workers in maintaining community spaces and creating customer confidence in safely enjoying a ‘hospitable’ environment. This article contributes to social science scholarship of embodied and emotional labour, hospitality and social reproduction.

## Introduction

In order to minimise the spread of the coronavirus disease 2019 (COVID-19), the UK government imposed its first national lockdown in March 2020, with Prime Minister Boris Johnson instructing people to ‘stay at home’ (BBC News, 2020a). Residents were only permitted to leave their homes to shop for essential items and to exercise once per day. Shops selling non-essential goods were closed, alongside schools, libraries, playgrounds, places of worship, hotels and all hospitality venues. Lockdowns exposed disparities across who was permitted, denied or urged to work, and under what circumstances (Dobusch and Kreissl, 2020: 711). Some service workers were required to continue as usual during lockdowns (e.g. facilitating takeaway orders) whereas other publics made use of these services while under mandate to work from home. When the phased reopening began in the summer of 2020, much of the media and political narratives about new COVID-19 restrictions and rising transmission rates were told through contention over the reopening of hospitality and the policy measures that followed.

The ‘Eat Out to Help Out’ scheme, for example, sought to increase hospitality revenue by offering a 50% discount to customers eating in venues in August 2020. A few weeks later, ‘the rule of six’ made it illegal for people to gather in large groups, including in hospitality settings; in the same month a 10 p.m. curfew was set for hospitality venues in England. By December 2020, businesses in some areas of the country were only allowed to serve alcohol indoors when sold alongside a ‘substantial meal’. The reopening of venues and the preparation of these spaces left hospitality workers responsible for making them ‘safe’ for the general public at a time when the judgement behind their use was in question. For instance, it was reported that only two in 10 adults were happy to have a sit-down meal in July 2020 (BBC News, 2020b), and by September 2020, fears were growing that the restrictions had been eased too quickly (McDonnell, 2020).

Despite the anticipation ahead of the lifting of the first lockdown, newspapers were filled with photographs of overflowing bars and complaints about irresponsibility. *The Guardian* proclaimed that ‘nowhere [. . .] has been more divisive than the pub’ (Williams, 2020). As an example of a particularly ‘cruel anomaly’, in September 2020 it was noted that you could go for a pint, ‘but you will be on your own when you first hear your baby’s heartbeat’ due to the prohibition against partners attending pregnancy appointments (Williams, 2020). Thus, for some, the pub became an example of a trivial pursuit with unnecessary risk, in contrast to other realms of life that were understood as more deserving but remained off-limits. The contrast became especially apparent as transmission rates started to rise following the first reopening, and pubs were positioned in conflict with schools. During the summer holidays in August, Professor Graham Medley, member of the UK Scientific Advisory Group for Emergencies (SAGE), suggested that new closures may be required ‘to enable us to open schools. It might come down to a question of which do you trade off against each other, and then that’s a matter of prioritising. Do we think pubs are more important than schools?’ (BBC News, 2020c).

As hospitality reopened, hygiene practices were central to COVID-19 safety guidance, with workers required to repeatedly clean premises, in some cases replacing the work of cleaning staff, in addition to their usual duties (Wood, 2020). Toilets were identified by the UK government as sites requiring particular attention, with the potential to put occupants at an elevated risk of transmission (UK Government, 2021). Businesses were therefore advised to clean toilets more frequently and to disinfect touchpoints and high footfall areas. Meanwhile, as people were permitted to meet again (with the rules emphasising outdoor socialising), the lack of access to public toilets emerged as a concern. The recurring lockdowns and reopenings of the hospitality sector laid bare the previous decade of austerity-hit public provisions and the growing reliance on commercial spaces (and the people maintaining them) for toilet facilities (Jones et al., 2020; Slater and Jones, 2018, 2021; White, 2021a). Hospitality has thus been used as a social and community infrastructure (Thurnell-Read, 2021a), substituting for declining public provisions and community services.

With these shifts in mind, this article shares findings from a research project that explored the experiences of workers involved in the cleaning and maintenance of hospitality settings and their hygiene facilities. We focus in particular on the embodied and emotional labour required of workers during the pandemic: from the heightened awareness and increased work of maintaining boundaries and instilling confidence in customers, through to the care and recuperation of bodies and personal lives at home. We demonstrate how hospitality work during the COVID-19 pandemic required new attention to corporeal practices and customer confidence, which we suggest constitutes a process of social reproduction. Drawing on the accounts of 21 hospitality workers in the UK, we bring together sociological literature on hospitality with scholarship on embodied and emotional labour. We seek to centralise the worker and the body within the context of, and scholarship on, hospitality.

## Situating Hospitality Work

Amid political narratives surrounding the (re)opening of hospitality, little attention was given to those working in the sector at a time when public-facing roles involved significant health risks. The already-fractured financial provision for hospitality workers means that job insecurity is prevalent, most often in the form of temporary shift work – where hours and pay can vary – and zero-hour or variable-hour contracts with low guaranteed hours (Baum et al., 2020; Thomas et al., 2020). Workers are often on minimum wage (Thomas et al., 2020) and in part-time work (Mai and Cominetti, 2020), and there is an overrepresentation of people of colour and those aged 16 to 24 (Mai and Cominetti, 2020). Systems of marginalisation are sustained within as well as between workplaces, with workers in the hospitality sector treated differently according to embodied intersections including race, gender and migration status (Alberti and Iannuzzi, 2020; Coffey et al., 2021).

Part-time workers, low-paid workers and sectors with higher rates of in-work poverty were especially affected by the pandemic (Joseph Rowntree Foundation, 2022). Research shows that those who had been struggling *before* COVID-19 were likely to work in precarious jobs or sectors that were hit the hardest by restrictions (Joseph Rowntree Foundation, 2022). Indeed, Baum et al. (2020) argue that COVID-19 has not so much changed poor working conditions in the hospitality sector as amplified them.

Hospitality workers tried to balance risks of virus control and financial (in)stability during the pandemic (Chen et al., 2022; Hadjisolomou and Simone, 2021). Given the difficulties for workers in the sector during this time (BBC News, 2020d), some looked for alternative work. Job insecurity and infection risk both contributed to workers in the USA leaving this sector (Chen et al., 2022). Such findings illuminate that government loans and grants rarely provided sufficient protection. In the UK, staff on furlough received only 80% of an already-low wage (with no tips) and were left without clarity around reopening. Insecure workers are nearly 10 times more likely to receive no sick pay (TUC, 2021), meaning they often have little choice but to continue working when they are ill or need to self-isolate, bringing risk to both their own and others’ health. Holwitt (2021) shows that the body is central to the transformations brought about by COVID-19 (see also Purnell, 2020), however there is little analysis that centres the body in relation to hospitality and COVID-19.

## Embodied and Emotional Labour

Body work scholarship develops sociologies of the body and of work, to explore the various roles of bodies at work. This literature emerged due to a tendency for sociological studies of work to overlook matters of embodiment (Gimlin, 2007; Shilling, 2003) and, similarly, for the sociology of the body to neglect issues of production, employment and labour (Gimlin, 2007; Wolkowitz, 2006). As Gimlin (2007) describes, a ‘body/work nexus’ was developed in light of these omissions to explore work relations and corporeality in new ways. Body work is a concept most readily applied to health and social care (Twigg et al., 2011) and beauty sectors (Black, 2004; Toerien and Kitzinger, 2007), where workers are paid to provide body treatments and therapies. While hospitality work has always been ‘embodied’, the significance of workers’ and customers’ bodies in this sector has heightened in the pandemic, given the new emphasis on disease transmission and risk.

We propose that the pandemic has required specific kinds of labour for hospitality workers. In some instances, this involved entirely new responsibilities compared with pre-pandemic times, and in other cases it was the same work but intensified. In keeping with healthcare literature, body work in this article includes hospitality work performed by staff on their own bodies as part of regimens of health, safety and well-being (Shilling, 2003), as well as ‘risk’ work carried out on the bodies of customers and other staff (Twigg, 2000). The aims of these interventions are multiple and, of course, in some cases intended specifically to create distance from the bodily in efforts to prevent the spread of COVID-19. For hospitality workers, this also includes bodily waste management or ‘dirty work’ – jobs that are often done by ‘the lowest paid, least regarded workers’ (Twigg, 2000: 391). Thus, body work can be understood as ‘ambivalent [and potentially demeaning] work’, fraught by power dynamics, ‘subordination and domination’ (Twigg, 2000: 391).

Further, we argue that pandemic body work also involves hospitality staff taking on roles of emotional management (Hochschild, 1983). Hochschild’s concept of emotional labour is most often interpreted as work carried out by staff on their own bodily and facial display, whereby their feelings are ‘economic commodities to be bought and sold’ (Wainwright and Calnan, 2002: 97). To achieve customer satisfaction and loyalty (Simillidou et al., 2020), this work is often a requirement of hospitality staff and explicitly enforced in employee guidelines. This entails faking and suppressing emotions, as well as exertions to actively feel the emotions expected (Hochschild, 1983). In hospitality, emotion management is understood as a particular imperative in staff’s handling of aggressive customers (Goussinsky, 2011) or unwanted sexual attention (Green, 2022), and has emerged as a key area of interest in organisational behaviour and customer service research (Goussinsky, 2011; Grandey et al., 2015; Simillidou et al., 2020). This growing acceptance of emotional work within organisational cultures may be indicative of a wider ‘affective turn’ in production (Reed and Ellis, 2020).

For some scholars, the requirement to perform emotional labour at work is unfair and indicates that staff are undervalued by their organisations, disrespected by customers and undermined by organisational policies (Grandey et al., 2015). Emotion management has been found to have a detrimental impact on some workers, creating emotional dissonance, a decreased sense of well-being and job burnout, particularly in workplaces where employees have limited autonomy (Goussinsky, 2011). Less attention has been given to the ways workers engage with customers’ emotions (see Hochschild, 1983; James, 1989; Kessler et al., 2015). In this article, we consider how pandemic body and emotion work in the hospitality sector is also designed to impact customers’ emotional responses: reassuring them about safeguards against COVID-19 transmission, and encouraging them to feel calm, safe and happy in the hospitality setting. The provision of a welcoming communal environment has always been a requirement in the hospitality sector (Thurnell-Read, 2021a) and workers themselves exercise collective emotional labour and sociality (Korczynski, 2003). However, we argue that the health and safety demands of the pandemic have been disruptive, requiring workers to ‘double down’ and over-compensate in order to restore a sense of pleasure in these settings.

There has been some deliberation over whether emotional labour should qualify as skilled work – particularly in roles traditionally understood as low-skilled (Payne, 2009) – with some researchers highlighting implications for how workers are paid, treated and viewed by their employers (Kessler et al., 2015). However, the skill involved in emotional labour is neglected and stigmatised due to its associations with women’s work (James, 1989). Furthermore, emotional labour as a form of social reproduction is naturalised, serving to hide both the value and the product of the labour (James, 1989: 22). Social reproduction describes ‘the activities that nurture future workers, regenerate the current workforce, and maintain those who cannot work’ (Hester and Srnicek, 2018: para. 1). As Bezanson and Luxton (2006: 37) describe, ‘both labors [paid labour and unpaid domestic work] are part of the same socio-economic process’. Traditionally performed by women for low or no wages, both in the household and the workplace, social reproduction work provides the everyday maintenance and reproduction of life (Hester and Srnicek, 2018). This may include caring for oneself and for others (healthcare, childcare, cooking), maintaining physical spaces (repairing, cleaning) and restoring or organising resources (shopping, washing clothes) (Hester and Srnicek, 2018).

While theories of emotional labour, body work and social reproduction are underpinned by gendered divisions and dynamics, they also lay bare broader relations of power, authority and status (Hochschild, 1983: 162) pertinent to low-paid, ‘frontline’ workers during a pandemic. In this article, we explore how COVID-19 public health guidelines have required hospitality workers to perform new social reproduction tasks to mitigate transmission, particularly un-/under-paid body and emotion work in the workplace and home. We argue this is indicative of the rising crisis of care (Hester and Srnicek, 2018) and the decimation of vital community provisions (Hall, 2020), which have been substituted by the hospitality sector. Before we turn to this analysis, we describe the research project on which this article is based.

## The Study

In December 2020, we began recruiting people who had worked in the hospitality sector at any point since the first lockdown of March 2020 (Jones et al., 2022). We advertised for participants whose roles involved cleaning, including the maintenance and monitoring of customer toilets. We shared our recruitment notice on social media platforms, including hospitality-related groups and local forums, and with relevant trade union contacts and mailing lists. Data collection ran until April 2021 and addressed participants’ experiences of national lockdowns, changing regulations and working in venues open to customers. Participants were invited to keep a flexible work diary after shifts or on non-work days over a two-week period, for up to two hours in total (Jones, 2022; White, 2021b). Diary guidance was provided with optional prompts, including details of a recent shift; perceptions of comfort, risk and safety; and any changes to cleaning responsibilities, toilet layouts and walking routes around their venue. Participants were able to select their preferred format, but all chose written description, including some supporting drawings, diagrams and maps. Diaries were diverse in their completion, such as in the level of description and reporting of tasks, through to the emotional and interpretative reflections.

Following the diary process, participants attended remote follow-up interviews with one of the four researchers. This included semi-structured questions as well as working as a ‘process of expansion’ (Zimmerman and Wieder, 1977: 491) from the participants’ diaries, allowing for mutual understanding, analysis and interpretation of their accounts. All participants were asked whether/how the pandemic changed their labour, particularly in terms of work quantity, equipment and responsibilities; the extent to which new tasks were monitored/assessed; their involvement or consultation in implementing new tasks; and the training and guidance they received. They were also asked about any changes to their feelings about work, their working/customer relationships and homelife. The four researchers undertook inductive thematic analysis by independently reviewing the diary and interview data, then identifying open and axial codes through consensus at team meetings.

A total of 21 hospitality workers participated. We collected demographic information using open-ended questions to allow for self-identification. This was optional and some participants chose not to respond. We spoke to workers in rural and urban locations in England and Northern Ireland, but no one from Scotland or Wales despite targeted recruitment. The majority of participants worked in pubs or restaurants, with some working in fast food outlets, bars or cafes, or across multiple types of venues. Most participants had a general staff role, but we also heard from three managers. Two participants described themselves as cleaners primarily. A few more women (57%) than men participated. Participants’ ages ranged from 18 to 65, with the largest proportion between 18 and 25 (43%). While 48% of participants self-defined as White British, we also spoke with participants identifying as White Irish, Black, Mixed Black, Mixed Race, Asian, Arab, White American, Indian and White Brazilian. Most participants identified as working class (33%) or middle class (29%), and two said lower-middle class. The majority of participants were heterosexual, and one-third defined themselves as bisexual, gay or lesbian. Three participants told us they had health conditions, although other experiences of impairment and/or illness were mentioned in interviews.

Institutional ethical approval was received from the University of Exeter. Much of the project’s design and implementation centred on careful ethical consideration of what it meant to be a precarious worker participating in research during a public health emergency. Knowing that many workers might be facing financial strain, it was important that participants were reimbursed for their time and contributions, including completion of the diary (£50), interview (£25) and group analysis workshops (£75). Participants were invited to review transcripts and to anonymise themselves and their workplace as they wished.

This was a collaborative project, guided by the principle that people working in or with the hospitality sector have expertise to benefit the research. An advisory group was formed for the study, composed of hospitality workers, trade union representatives, local campaigners and work researchers. We held three online meetings to consult our six advisors on ethics, recruitment, methods, research questions and communication of findings. Participants were also invited to one of two two-hour online participatory analysis sessions led by the researchers in June 2021. Twelve participants attended. We discussed a thematic summary of findings distributed to participants prior to the session, asking for their perspective on the key themes; the context, background, potential causes of, and solutions to, the work issues raised; and any important areas that we may have omitted. Insights from the advisory group and participatory analysis sessions have contributed to this article (Jones et al., 2022). Given the changing nature of the COVID-19 pandemic and its regulations, the accounts provided in our research are time-sensitive and participants’ views may have changed since data collection.

## Findings

### Overwork, Fatigue and Boundaries

When this project began in Autumn 2020, much of the news media focused on the reopening of the hospitality sector and the introduction of COVID-19 regulations. These included policies on face coverings; the two-metre rule; closure of customer toilets; and the NHS Test and Trace system, which required staff to collect customers’ contact details upon entry to the venue. As the pandemic progressed, different challenges arose when managing new requirements while still creating a ‘hospitable’ environment. Participants described efforts to create and maintain bodily boundaries to protect customers and themselves from catching and spreading COVID-19, while managing the expectations of intensified tasks.

Negotiated boundaries and transgressions of space were further magnified by additional COVID-19 regulations implemented in September 2020, requiring venues to operate table service only. As one participant, Alesha, described, this prevented her from ‘being able to escape contact by being behind the bar’ or being shielded by newly installed Perspex screens. Participants also discussed overcrowding in venues, and their responsibility to manage the flow of customers in confined areas. Thus, managing their own bodies, and the distance between others, became ongoing physical labour (Gimlin, 2007). Participants not only felt exposed because of the risk of catching COVID-19, but also highlighted altered relationships with customers due to new regulations. One participant, Dee, wrote in her diary:

I feel unsafe when I have to stand at the entrance to ask people to do track and trace, as you have to ask everyone that comes in – and a lot of customers get really impatient at the fact that they have to do it.

Dee also discussed how customers sometimes intruded on her personal space:

It was strange that we were close to people taking someone’s order. It made sense to me that really, we are sanitising and as much as we can we kept our big doors open so we could ventilate the place, but . . . As it went on, masks became a thing indoors and you got a bit more wary of people. You would always have to be like, ‘No, sit down.’ They get friendly and they want to put an arm around you or chat to you this close. ‘No, you can say it from over there.’

Unwanted sexual attention may have already been familiar to bar staff, particularly women, whose ‘professionalised’ knowledge of emotion management and physical distancing to negotiate inappropriate customers is learned on the job (Green, 2022). However, the embodied labour in politely navigating customers during the pandemic also came with an intensification of work, particularly the increased requirements of cleaning. The heavier workload was often described as exhausting, with time compressed or extended as participants reported staying late to work over their shift, being unable to chat to customers as they would usually or having to spend more time cleaning spaces perceived as ‘high-risk’, such as the toilet. For example, Pooja noted the ‘constant cleaning’ in her diary:

There were 15-minute timed alarms which went off meaning we had to disinfect different areas of the toilets, bar area . . . We had to sanitise down all bathroom surfaces and touch points along the corridors when a 30-minute timer went off. We also had extra cleaning duties before open and after close.

Despite this additional labour, Pooja noted that staff received no extra time to complete the work and breaks were not increased. She explained that it had been ‘physically really exhausting, because even though they were small tasks, they were constant’.

On top of the intensification of workload and the resulting physical exhaustion, there were further embodied effects such as aching legs and feet from the increased work of table service, as well as overheating and difficulties due to wearing face coverings. Many participants also highlighted that their hands had become sore as a result of the cleaning regimes, hand sanitising and washing. Evidence of the strain of hospitality work on the body (Gimlin, 2007) was visible in broken, burnt and sore skin. Pooja explained that her hands were physically marked by her work:

I have quite bad eczema on my hands and . . . it’s a really strong alcohol [and] stingy on my hands . . . even though we had gloves on, you would do all the cleaning but then you would wash your hands after and the more I would wash my hands, the more it would dry out. My right hand got so sore I had to get a special prescription for a steroid tape that you put on the sore bits to protect them.

The wearing of face coverings was another requirement by which hospitality workers came to perform embodied and emotional work. Despite the reopening of hospitality in July 2020, use of face coverings was only made compulsory in the sector in September that year. This was a cause for concern for many of our participants, including Les who began wearing one before it became a rule. Wearing face coverings to suppress COVID-19 transmission, and the regulation of customers’ and colleagues’ use of coverings became an ongoing form of body work and emotional labour due to the conflict sometimes provoked by policing new laws. Wearing face coverings also came with challenges as it limited workers’ ability to continue the expected emotional work and ‘hospitable’ service. Through the hiding of the mouth – akin to Hochschild’s (1983) conceptualisation of ‘facial display’ – the workers’ visual presentations of emotions were concealed. While this often brought difficulties, as we explore below, it could also have valuable outcomes for workers, such as waiving the obligation to perform the ‘surface act’ of forcing a smile or feigning positive emotions (Simillidou et al., 2020).

Hospitality workers accepted the benefit of the face coverings for public health purposes, and some disclosed a discomfort in their interviews about complaining about the coverings given their potential protective value. Nevertheless, the responsibility of wearing and enforcing their use was significant, alongside the associated body work and physical impacts. Several participants highlighted the difficulty of wearing face coverings in particular areas, such as hot kitchens and rooms with dishwasher steam, where breathing became challenging. As Les noted, ‘when you’re on a grill, flipping burgers and doing stuff, it’s hot behind the mainline. With a mask on top, it’s uncomfortable.’ Chris explained how his mask affected his attention to customers’ access needs:

[My manager] described it as either you can’t breathe or you can’t hear [. . .] It is difficult doing the waiting with a mask on, particularly because we have a lot of elderly people in and a lot of people who try to lip read or at least augment the sense with lip reading. And that is obviously impossible with a mask on. So I switched to wearing a mask when I was on the bar, because that is more effective because of aerosol contagion and everything, but when I was on the floor I wore the visor instead because then people can see your mouth.

Chris’s account highlights not only the physical impact of face coverings but also the labour of finding new ways to ensure effective ‘face-to-face, voice-to-voice contact with customers’ (Gimlin, 2007: 361). Switching between types of face coverings was deemed necessary for aiding accessibility in the communicative, sensory and emotional work of hospitality, providing good and caring ‘customer service’ and cultivating relationships. The government’s 2020 public health campaign, ‘Hands. Face. Space.’, captures forms of body work – apparent in marked hands, covered faces and distances created through Perspex screens and stickers. As we have illustrated, individualised exertion by workers was required to protect and maintain each of these three sites.

### Forging Confidence in Hospitality

In addition to the harmful physical consequences described above, a lack of clear government instructions meant that discretion about cleanliness and risk was often left with each worker, individualising responsibility and creating a new burden of guilt when work was especially busy and tasks had to be skipped. One participant, Sara, recalled: ‘It was really a bit chaotic to begin with because there were no set rules, unfortunately, so it was all just, like, not even weekly, just biweekly sort of verbal reminders, or just telling us to do deeper cleans.’ Many participants felt demoralised and overwhelmed by the degree of responsibility they carried due to risk-management work. They also highlighted the ways that pandemic risk assessments and ‘COVID-secure’ public health guidance were vastly inadequate or misleading. Their lack of trust in the efficacy of the guidance produced a moral burden about their own role in transmission.

Political and social trust in the government and COVID-19 health policies has a critical relationship to public compliance, risk perception and mortality rates (Devine et al., 2021). However, public confidence in the UK government’s pandemic strategy was precarious from an early stage and subject to various setbacks. In February 2020, after announcing the first COVID-19 death of a British national, Prime Minister Boris Johnson explained that the ‘best single piece of advice we can give’ is to ‘wash your hands for 20 seconds or more’ (BBC News, 2020e). While this message was later replaced by a more expansive notion of transmission, hand-washing and disinfection have nevertheless remained central to the campaign to stop the spread. The public and service workers have been responsibilised through this emphasis (King et al., 2021). At the time of writing, in summer 2022, visible provisions and signage supporting these low-cost personal protective measures can still be found in venues across the UK, but the government has done little to monitor or regulate environmental improvements to address aerosol spread, such as ventilation and air infiltration.

Amid the confusion and anxiety around risk, various COVID-19 commentaries have declared the emphasis on cleaning to be ‘hygiene theatre’, described in *The Atlantic*, for example, as a ‘bonanza of pointless power-scrubbing’ (Thompson, 2021). The concept of ‘hygiene theatre’ emerged in some of our participants’ descriptions of their work. Leah, for example, reflected that ‘it’s an airborne virus, but people want to see you cleaning surfaces more. It doesn’t matter how clean this table is, you could just look up and inhale coronavirus.’ Similarly, Chris reflected that cleaning had begun to feel ‘more like you are doing it just for the sake of the look of it’. He added that the public health potential of cleaning work was also undermined by a profit framework that prioritised ‘the needs of the business’. Chris explained that the assistant manager advised him that ‘if there’s a queue of drinks to be done then that’s more important than making sure that the toilets are to be cleaned every half-hour’. Chris reported that the manager agreed with this in theory but pointed out that:

It’s actually really good for repeat business if customers think that we are really, really focused on making sure it’s clean. That reassures people and they may be more likely to want to come back because they feel it’s more safe.

In either case, Chris realised that it was ‘all understood in terms of the business case’, with cleaning playing a contested role: it could be a hindrance and/or a foothold for more custom, depending on your perspective.

Both Chris and Leah underlined the presumed importance of their workplace ‘appearing’ clean to the public, and for cleaning acts to be observed. In doing so, customers may have been reassured that staff were exerting control over transmission risk, even though the benefits to public health may have been questionable. The emphasis on visibility could also detract from less visible forms of aerial risk management – the spread of the disease through air and respiratory droplets – which are more difficult and potentially expensive to control in hospitality settings. Cleaning, as described by Leah and Chris, focuses on providing a sense of *emotional* security and comfort to customers, so that they will stay longer and continue to return. This type of emotional labour may be new to many hospitality workers whose pre-pandemic work was not perceived as ‘risky’ to the same degree, but this is nevertheless complex and skilled work (Kessler et al., 2015), which is often ‘unrecognised, unrecorded, and unrewarded’ (James, 1989: 39). Staff were not always convinced of the validity or efficacy of this form of risk management, which in turn created a moral burden, additional to the physical costs of carrying out the body work.

## Domestic Care Work

We have shared participants’ descriptions of the substantial amount of bodily and emotional discomfort, pressure and work intensity. Work responsibilities and customer care also pervaded their private home lives. While some of this body and emotional work was required by employers or the government, other aspects could be understood as ‘voluntary’ measures to make work feel more manageable in difficult circumstances, to help workers feel safe and protect others around them. A sense of accountability was dominant in many narratives. Ronald, for example, explained how he would ‘[keep] an eye on advice’, ‘[pay attention] to what others working in pubs were doing’ and ‘browse on google and read articles about good practices to tell the boss or share with my mates’.

As a result of poor and confusing guidelines, and the heightened anxiety many of the workers felt about their role in the spread of the disease and their duty of care to cohabitants, participants told us about the extensive and routine body work they carried out at home before and after work. This included regular handwashing, carefully removing and washing clothes and face coverings, showering or bathing after every shift, regularly cleaning and cutting nails, and taking special care around people living in the same home. Caper described her after-work routine: ‘I would wipe down [the car] as much as possible for anyone else. I wasn’t allowed to touch anything when I came back in.’ She also recalled discussing with a colleague their methods to ‘avoid touching our partners’.

Although not all of the domestic body work participants listed was explicitly required by employers, staff felt a social responsibility to ensure they took appropriate measures to mitigate transmission risk and therefore brought their duties into the privacy of their homes. This was experienced as a moral burden for some due to the social values accorded to transmission and protection, and public debates about the risks of hospitality. In some cases, employers made this labour more onerous by refusing to follow health and safety guidance or failing to provide adequate protections or accommodations. Pooja, for example, explained that despite asking for another uniform, she was only given one work shirt. She commented:

Sometimes I wasn’t getting home until after 1 am and I would have to shower quickly when I got in because I won’t sit down or anything until I’ve showered. I’m also then supposed to be cleaning my shirt for work the next day. Unfortunately, I wasn’t able to on some days because the next day I was working again on open [the shift opening the venue] and there literally wouldn’t have been time. So it’s pretty disgusting but I had to wear the same shirt the next day because they wouldn’t give me a second uniform.

In addition to measures to reduce transmission, domestic work was also required as a way of recuperating from the protective COVID-19 measures and work intensity described above. For example, Lily spoke about how her dry and painful hands were still recovering months later. She noted, ‘I even considered buying these gloves, and you slather moisturiser on and then you wear these gloves. I’ve got about ten hand-creams.’ This pre- and post-work unpaid labour was significant in some cases and was especially difficult for those with other caring responsibilities. As acts of largely unrecognised care, this work shares similarities with Hochschild’s (2012) ‘second shift’ or ‘double burden’, a concept referring to the extra time women often spend on chores, childcare and other care work in heteronormative nuclear-family households, in addition to their paid jobs.

For some participants during the pandemic, the domestic body work – or ‘protective’ and recuperative labour – that we have described may constitute a ‘*third* shift’ added to the other domestic labour they already provide (Gerstel, 2000). As Hochschild has shown, this additional work has various negative impacts, including limited recreation time, less sleep, physical illness and stress. In the hospitality sector – a relatively young, but diverse workforce – these repercussions will affect some more severely than others, due to occupational and domestic hierarchies and inequalities across gender, race and other intersections. Similar to the emotional labour illustrated above, body work, which takes place in the home in private, or at work but in excess of agreed/paid hours, is rendered invisible and undervalued by others. Like most feminised labour, this labour is subordinated – as James (1989: 40) argues – as ‘peripheral’ and ‘merely “support” work’. Thus, Hester and Srnicek (2018) stress the need to agitate for a ‘post-gender’ society as part of the struggle against work: rethinking living arrangements and notions of ‘the family’, and challenging the binary gender system, which has informed naturalised divisions of labour and value. These systems, they argue, are intimately intertwined – especially during the pandemic when normative assumptions about ‘the household’ became a tool for the UK government to contain risk.

## Conclusion

This article explores areas where there is currently a lack of theoretical emphasis. Body work and emotional labour scholarship has tended to focus on work on or with the body in healthcare and beauty sectors, however we sought to prioritise the emotional and embodied experiences of hospitality workers, and their prominent role in broader COVID-19 ‘pandemic work’ and social reproduction. While work in this sector already required embodied and emotional labour, we provide original insight into how COVID-19 guidance has expanded and intensified the body and emotion work expected of and performed by staff. These responsibilities are especially significant in an underpaid sector, with a disproportionate number of marginalised workers.

First, participants described heavier workloads due to the requirement to enforce and regulate safety by undertaking new hygiene regimes and preserving bodily boundaries and distance. This had damaging physical ramifications and was illustrated through new workplace policies such as table service and timed alarms to schedule cleaning and maintenance. Second, participants were required to provide emotional reassurance to customers and foster confidence in hospitality settings. Intentionally visible cleaning work was prioritised by employers to protect revenue, compelling staff to discount their concerns about the (in)efficacy of disinfection. Third, these physical and emotional demands of participants extended beyond the workplace: from repairing their own bodies and washing uniforms and masks, to exercising caution within the home and protecting cohabitants. These accounts revealed the role of care within and beyond the hospitality setting – often unseen and unacknowledged – in the collective attempt to keep COVID-19 rates low.

We note, however, that state provision for social reproduction work has been stripped back (Hester and Srnicek, 2018), and a lack of resources to support dependent others has resulted in a crisis of care in public and personal reproductive labour (Hester and Srnicek, 2018). Meanwhile, austerity measures have significantly reduced vital community provisions (Hall, 2020) such as public toilets, which has led to private-sector buildings being used as insufficient substitutes (Jones et al., 2020; Slater and Jones, 2018, 2021; White, 2021a). Owing to the decimation of these social infrastructures, we illustrated that care work has been delegated and integrated into other spaces; in this case, hospitality duties and domestic tasks as additional and personalised reproductive labour. This situation was especially conspicuous and arduous during the pandemic, where service and other low-paid workers were tasked with new responsibilities (‘pandemic work’) to ensure public safety, sociality and sustenance. The body and emotion work we have detailed – and the new techniques and requirements involved in such labour – were transferred into existing hospitality roles, surplus to agreed hours and duties.

The workers in this research described a tangled relationship between the private and public parts of their working lives during the pandemic. While additional body work in the home may have provided some relief and offered workers a way to cope, we have shown how this labour is also vastly undervalued and thus ‘hidden’, particularly as feminised work in domestic spaces is routinely discredited. As Fraser (2017: 23) comments, ‘the capitalist economy relies on – one might say, free-rides on – activities of provisioning, caregiving, and interaction’, but does not ‘[accord them] monetized value and treats them as if they were free’. Nevertheless, through this additional labour, hospitality workers may have played a fundamental role in preventing the spread of COVID-19 – in other words, ‘staying alive and helping others stay alive’ (Hester and Srnicek, 2018: para. 1). This article thus contributes new and innovative ways of conceptualising the relationship between social reproduction, the rising crisis of care and the hospitality sector. Further, our findings illustrate a broader societal trend, whereby responsibilities are shouldered by hospitality workers due to inadequate state and public provisions for social care and public health.

This responsibility was a substantial moral burden for participants. The ‘virtue’ of the protective work becomes part of its pull; as was once argued in the domestic labour debate, we may need to contest ‘at once the invisibility’ of this work and ‘its moralisation’ in order to ‘redress both its devaluation as work and its overvaluation as labor of love’ (Weeks, 2011: 124). Some participants argued that they should be receiving ‘hazard pay’ in recognition of the elevated danger their work now posed to them. We would argue that extra time at home spent mitigating these dangers could also be financially recompensed with a real living wage. Our focus on socially reproductive work indicates a need to address our under-resourced care system and austerity-hit community provisions, while paying attention to the ramifications of such on other sectors and workers. This requires a radical re-working of service relations and reproduction, including the gendering of labour and its value, and the fluid and complex dynamics between life and work. There is a need for future research to consider intersecting inequalities within the hospitality workforce, and the uneven impact and meaning of these changes on a diversity of workers.

Despite public disunity over the safety of hospitality during the pandemic, its importance as a community infrastructure has been defended emphatically (Thurnell-Read, 2021b). However, little has been said about the important role played by hospitality workers in sustaining such sociality – workers who were already undervalued, overworked and often otherwise socially marginalised. As argued by the Notes from Below Collective (2020: 186), ‘corona did not fundamentally draw new separation lines within the labour market but drastically increased existing divisions and inequalities’. Similarly, the invisible and unrecognised status of reproductive labour was nothing new, even though the extensive body and emotional work imposed on hospitality staff was different or harder than before. The pandemic illustrated that the state is unwilling to take responsibility for the health and safety of hospitality workers, or recognise the insecure nature of such work and its relationship to existing inequalities (Jones et al., 2020, 2022). A collective response through trade unions and workers’ movements is therefore necessary to make this progress.
